# A Call to Action for Qualitative Behavior Analysis

**DOI:** 10.1007/s40617-025-01096-3

**Published:** 2025-10-06

**Authors:** Victoria Burney, Ebonee Hodder, Clare M. McCann, Sehar Moughal

**Affiliations:** School of Psychology, The University of Auckland, Auckland, New Zealand

## Abstract

The convergence of many shifts within and outside the field of behavior analysis have led to an important conclusion: now more than ever before, qualitative behavior analysis as a distinct branch of behaviorism is warranted, useful, and necessary. This article explores what qualitative behavior analysis could be, and do, within the field of behavior analysis. An outline of how qualitative behavior analysis can fit within the broader field is provided. Ways in which qualitative behavior analysis can be considered consistent with radical behaviorism are discussed, alongside a consideration of tenets which discriminate qualitative behavior analysis as a unique branch of the broader behavior analytic field. In advancing a call for qualitative behavior analysis, we invite the field to consider and discuss how, and why, a qualitative branch to our science might bring value, and ameliorate some of the big issues facing behavior analysis today.

## Preamble

The authors of this article approach this topic as scholars trained in behavior analysis (with the exception of one author who is a speech-language therapist with an appreciation for the utility of behavior analysis), who research and practice in applied settings and contexts. Our collective learning histories (including our training) have resulted in the value and prioritization of the application of behavior principles in areas of social significance, and we aim to align our practice with social validity, social justice, and culturally responsive lenses. We are all engaging with qualitative approaches as part of our research scholarship and appreciate how these approaches offer unique, interesting, and important, elements to our applied behavioral work. Our roots are firmly in the application of behavior analysis. Resultingly, this article considers the opportunity and potential of a distinct branch of behavior science—qualitative behavior analysis—as we understand this from our own perspectives and learning histories as applied behavior analysts. The conceptualisation of qualitative behavior analysis which we offer in this writing is intended to embolden the broader field of behavior science to consider how this might fit, or align, with their perspectives and practice of behaviorism. We hope this will generate debate and discussion across the broader field, as a starting point for considering a novel subsection of behavior analysis.

## Contextualising Qualitative Behavior Analysis

The invitation to broaden research approaches in behaviou analysis is not new. Skinner (1974) proposed that behavior analysts should select methods of inquiry based on the questions being investigated, rather than holding any specific methodological preoccupation. Other scholars have touted the value of different research methods (as compared with small-n design) to expand understandings of emerging topic areas (Malagodi, 1986; Neuringer, 1991), provide compelling evidence of the effectiveness of behavior analytic interventions (Dowdy et al., 2021; T. Smith, 2012), and further the dissemination of behavior analytic efforts (Friman, 2021). As early as the 1990s, behavior analysts posited that qualitative research methods, used well, could progress understandings of social validity in behavior analysis (Schwartz & Baer, 1991), and support examination of important behavioral topics from novel angles (Schwartz et al., 1995; Schwartz & Olswang, 1996).

Fast forward to here and now, where appeals to broaden investigative methods in behavior analysis have gone largely unheeded (Rader et al., 2021), as the context surrounding the field has shifted considerably (DeFelice & Diller, 2019; Penney et al., 2023). The exponential growth of behavior analytic applied practice (coupled with growing pains around increasing commodification and financial imperatives, maintaining high quality services, and providing effective supervision; Penney et al., 2023), alongside sweeping societal shifts in equity and diversity spaces (see Gingles et al., 2022; Zarcone et al., 2019, for a discussion), place the field of behavior analysis at a crossroads, facing a time of critical reflection and necessary change.

Increasingly urgent appeals to re-align applied behavior analysis with principles of compassion (Kelly et al., 2021; Marchese & Weiss, 2023; Taylor et al., 2018), alongside bids to practise humility in behaviorism (Kirby et al., 2022), challenges to embrace interdisciplinarity (Coy et al., 2024; Friedman & El-Roy, 2024) and calls to act in allyship with the neurodiversity movement (Mathur et al., 2024), have all shifted qualitative approaches back into focus for behavior analysts. The field is primed to examine how qualitative methods might afford us increased opportunity to meet our long-held ambitions: to shift behavior (of individuals, groups, and society most broadly) in meaningful ways, informing quality of life and social justice efforts (Heward et al., 2022; Pritchett et al., 2022; Schwartz & Kelly, 2021). Now is the time to revisit the place of qualitative approaches within behavior analysis.

Inclusion of qualitative methods in behavior analytic research offers potential. However, given the intersection of various socio-political movements facing the field, it may be time to think bigger and go further: is there space for qualitative behavior analysis as a distinct, but embedded, branch of behaviorism? The establishment of qualitative behavior analysis (an approach with qualitative assumptions and imperatives at its core) may help us to move forward as an inclusive, progressive science (Leaf et al., 2023; Pritchett et al., 2022). This article aims to outline qualitative behavior analysis as a discrete subset of behaviorism: how it could be conceptualised and situated within the field, and what it might offer behavior analysts. Possible tenets of qualitative behavior analysis are provided, alongside consideration of next steps to establish qualitative behavior analysis in service of alignment with behavior analytic values; to alleviate suffering and promote equity for individuals and communities (Dettmering & Hodzic, 2024). The article outlines a novel way of considering qualitative behavior analysis within our field, calling for further consideration and discussion of this proposed approach within our scholarship.

## Qualitative Behavior Analysis AS Behavior Analysis

Aligning qualitative behavior analysis as distinct from, but philosophically consistent with, the wider field of behavior analysis, is critical to the utility of this approach as part of our science. Notwithstanding apparent disparity in underlying assumptions, and topographical departures from our research ‘norms’ (Burney et al., 2024), qualitative behavior analysis can cohere with philosophical underpinnings and practical dimensions held as central to behavior analysis.

Qualitative behavior analysis can be seen *as* behavior analysis when interrogating the philosophical assumptions underlying the field. Importantly, qualitative approaches to behavior analysis can align with closely held (but often minimally described; Morris, 1988) ontological and epistemological positions inherent in radical behaviorism; the dominant philosophical position in behavior analysis. In unpacking the assumption that behavior analysis exclusively operates from a ‘monolithic psychological model’ driven by positivism, Ruiz (1995, p. 162) discriminated between methodological and contextual roots for behavior analytic ideas of knowing, with the latter aligned best to Skinner’s conceptualisation of how behaviorists ‘know’ (see Morris, 1992, 1993). Separating Skinner’s radical behaviorism from that built on Watson’s methodological behaviorism (see Hayes et al., 1988, for commentary), Ruiz (1995) argued that contextualist views espoused by Skinner align behaviorism more closely with worldviews or epistemologies which are generative, critical, and acknowledge the constructed and partial ways of ‘knowing’ about organism and environment interactions. From this perspective, radical behaviorism may allow for qualitative investigations, by being consistent with more contextualised, critical, interrogative, and intersectional positionalities typical of a qualitative lens (DeFelice & Diller, 2019; Ruiz & Roche, 2007).

Another widely held philosophical perspective within behavior analysis, that of functional contextualism (Biglan & Hayes, 2015; Hayes et al., 2001), also shares worldviews which are consistent with qualitative approaches. Aligned with characteristics of Pepper’s (1942) contextualism, Hayes (1993, 1997) explains functional contextualism as a way of seeing behavior analytic endeavours as relativistic, rejecting a notion of ultimate ‘Truth’ (out there to *find out* and report on), and positioning the goals, and actions, of behavior analysts in research as pragmatic, personal, subjective and informed by imperatives of the researcher (Barnes-Holmes, 2000; Hayes, 1993; Ruiz & Roche, 2007).

**Fig. 1:**
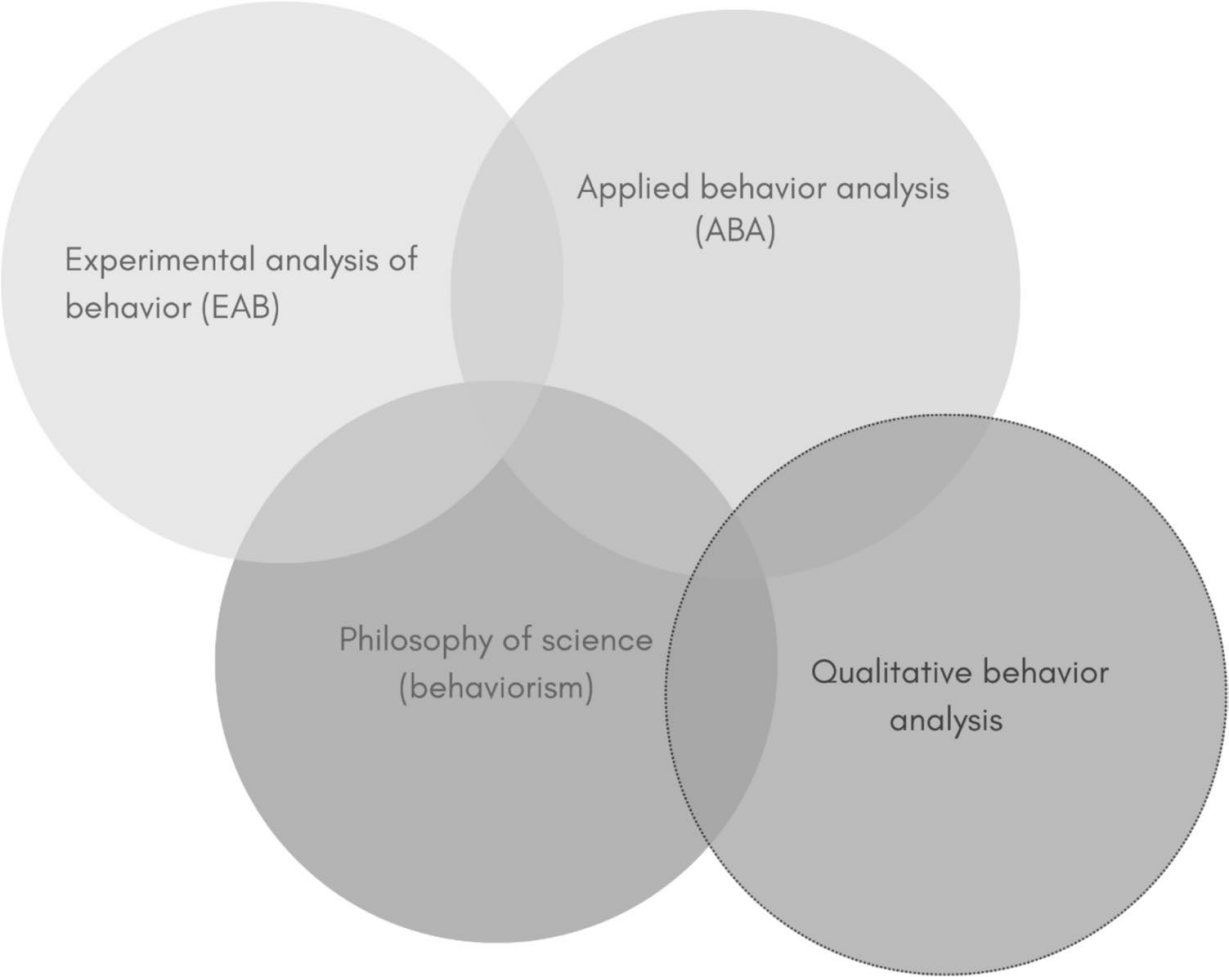
Venn diagram showing the relations between branches of behavior analysis

Holding the position that contextual radical behaviorism, and functional contextualism, cohere with constructivist perspectives where ‘the knowledge we create through scientific inquiry is relative, contextual, complex and subjective’ (Ruiz, 1995, p. 172)—and no universal truth can exist beyond the situational truth available to a researcher from their perspective (Jaeger & Rosnow, 1988; Madill et al., 2000)—means finding fit between the philosophical commitments of behavior analysis, and qualitative behavior analysis. From this viewpoint, qualitative ways of knowing are not antithetical to behavior analytic investigations but instead can be seen as consistent. Indeed, aligned with this argument, qualitative behavior analysis could be conceptualised as related to, but distinct from, the established strands of scientific endeavour comprising the field (specifically, philosophic accounts or behaviorism, experimental science (EAB), and the application of the science (ABA); Araiba, 2024; Michael, 1993). The potential conceptual links between qualitative behavior analysis and existing branches of behaviorism (most notably between the philosophy of behaviorism, and the applied ‘arm’ of the field) are illustrated in Fig. 1. Notwithstanding ongoing debate within the field around how subdisciplines of behavior science cohere and interrelate (see Araiba, 2024; Pierce & Epling, 1980; Poling et al., 1981, for discussion of these tensions), qualitative behavior analysis, as conceptualised in this article, holds practical and theoretical ties with branches of behaviorism and applied behavior analysis.

Qualitative behavior analysis can be understood *as* behavior analysis when interrogating ways that qualitative research is congruent with the seven dimensions of applied behavior analysis (Baer et al., 1968, 1987) that form the ‘bedrock’ for our applied science (Penney et al., 2023) and discriminate high quality behavior analysis from other psychological or scientific endeavours. Again, a branch of qualitative behavior analysis is conceptualised as having links (or crossover) with applied behavior analysis (ABA), while holding discrete features which position it as a unique branch of behaviorism (see, Fig. 1).

Summarised briefly, qualitative frames can be *applied* (focusing on meaningful domains for individuals and society), and *generalisable* (carefully extending insights to other, consistent environments). Increasingly, qualitative research endeavours are focused on rigour (using different measures to those common in behavior analysis), transparency, and congruence between assumptions, methods, and outcomes of research (see Braun & Clarke, 2024a, 2024b, for a discussion). In these ways qualitative approaches can meet the *analytic*, *conceptually systematic*, and *technological* dimensions necessary for behavior analytic research. In measuring verbal report (or private events), qualitative approaches can align with (if not completely satisfy) the *behavioral* dimension (see Baer et al., 1987; Burney et al., 2023, for a discussion of this perspective). Importantly, when considering possible functions of ‘building understandings or ‘gaining insight’ driving research, qualitative approaches may be the most *effective* (or efficient) technology available to analysts, satisfying this final dimension.

Fundamentally, qualitative behavior analysis presents an approach that offers meaningful opportunities to unpack the field’s ontological and epistemological positions and expand understandings of research in line with these. Qualitative behavior analysis can drive research and practice in exciting new directions, which are not only informed by, but deeply embedded in, the core philosophies and dimensions of the science. Importantly, qualitative behavior analysis provides an avenue for the principles of behaviorism to be realised through research and practice, in ways consistent with the dimensions of applied behavior analysis and additive for the field: promoting approaches that make visible relatively ‘invisible’ private events which precede, accompany, and explain, a variety of other behaviors, contexts, and environments, important to the field of behavior analysis and its continued growth (Hayes & Wilson, 1993).

## Tenets of Qualitative Behavior Analysis

Exploring what qualitative behavior analysis offers the field involves identifying elements that define qualitative behavior analysis, and that delineate this approach from other behavior analytic avenues. Borrowing from the conceptual work of QuantCRIT scholars (Castillo & Strunk, 2025; Strunk et al., 2024) and aligned with the principles outlined by Kelly et al. (2021), the following section describes four tenets, offered as a starting point for conceptualising qualitative behavior analysis.

### Function is Key

Just as a function-based perspective drives the selection and implementation of other behavior analytic activities (including assessment and intervention), so too should the function of the investigation drive the selection of specific methodological tools or research approaches in qualitative behavior analysis. When the purpose of research is to: advance knowledge of topics which sit at the fringe of behavioral conceptualisations (Critchfield & Reed, 2017; Dixon et al., 2018), to understand a situation or phenomenon from the perspective of those with direct experience (Neuringer, 1991), or to broaden viewpoints on an issue or context beyond what is discretely observable for a behavior analytic researcher (Fawcett, 1991), qualitative ways of researching might best serve these functions. Rather than dismissing qualitative approaches as secondary to other, more ‘rigorous’ research methods (P. Smith & Little, 2018), qualitative behavior analysis holds as a principle that function-based research approaches will generate the most useful and relevant research outputs, to further the reach of behavior analysis in valued directions (Ruiz & Roche, 2007). Notably, if the function of a specific investigation is to investigate a social phenomenon (of which behavior analytic scholarship has only nominal insight), behavior analysts may choose qualitative approaches which allow learning by listening to the narratives of people and considering the experience from this perspective (Pritchett et al., 2022; Taylor et al., 2018), over other ways of ‘knowing’ or other methodological tools.

### Data Are Not Neutral

A core tenet of qualitative behavior analysis is recognition that, rather than viewing data as objective, neutral, and free from bias (Castillo & Gillborn, 2023), all data are subject to processes of revision and interpretation by researchers, which preclude objectivity and negate assumptions that data can ‘speak for itself’ (Keller, 1995). Although behavior analytic research has long strived for objectivity through use of quantifiable data collection approaches, such investigative objectivity has never been realised (for a consideration of this approach in behavior analytic literature, see: Sidman, 1960). Indeed, researchers in behavior analysis reliably choose what questions to ask of whom, where, how, and for how long to measure, what data to present, and how to interpret these data (Pritchett et al., 2022). In this way, data are never ‘neutral’ but the product of the learning histories, values and priorities of researchers. Qualitative behavior analysis, in alignment with radical behaviorist and constructivist perspectives, embraces the inherent subjectivity of all research investigations, providing opportunities to research in ways that bring such assumptions to the forefront (Kelly et al., 2021; Schwartz & Kelly, 2021). Further, qualitative approaches in behavior analysis provide analysts with tools to identify, measure, and reflect on their own covert (or private, non-observable) behaviors (Neuringer, 1991), which shape (or inform) the way they undertake activities of behavior analytic research and practice. This tenet expounds the need for qualitative investigations in behavior analysis to be ‘open about the backgrounds and expertise of the researcher’ as a way to ‘reflect on, understand and address their position/s in society and any potential biases and blind-spots’ (Castillo & Gillborn, 2023, p. 3). One way this can be exemplified in qualitative behavior analysis is through the inclusion of positionality statements (see Holmes, 2020; or Savolainen et al., 2023 for a broader discussion), to contextualise the research and researchers for the audience.

### Social Validity at the Centre

Closely aligned with appeals for compassionate and collaborative approaches within behavior analysis, this tenet outlines that qualitative behavior analysis is inherently concerned with social validity and furthering social justice. Where other approaches to investigation in behavior analysis afford researchers the scope to set the agenda for investigative work (be this to gain insight into behavioral mechanisms, translate knowledge from experimental to applied settings, or test behavior analytic technologies), qualitative behavior analysis prioritises giving agency back to those at the centre of the research. In practice, this principle is evidenced by working with and alongside others, rather than a patriarchal ‘doing on and for’ others within the space of research—even when such research is centred around evaluating the social validity of behavior analytic intervention efforts (Melton et al., 2023). Penney et al. (2023) describe this approach as a ‘partnership’ rather than a process where behavior analysts ‘drive’ the research programme. Ultimately this repositioning of expertise helps to ensure that the processes and products of research are meaningful and useful for those who are involved in the investigation, not merely for the behavior analysts involved in the inquiry (Schwartz, 1991; Schwartz et al., 1995). By applying a social validity lens to selecting and defining research activities, qualitative behavior analysis can both further the social acceptability of behavior analytic research, and further behavior analytic accounts of social relevance and importance of work produced within our field (Leko, 2014). This tenet requires analysts to consider ‘who benefits from this work?’ (Patton, 2002) when positioning a qualitative behavior analytic investigation, and to consider the ecological validity of prioritising the concerns of stakeholders in setting the research aims and parameters (Fahmie et al., 2023).

### Reflectivity Matters

Crucially, qualitative behavior analysis implores analysts to interrogate their own behavior and contingencies. This involves building awareness of our own behaviors, and reflecting on how our behaviors align with, or diverge from, the aims and values driving the investigation (Dettmering & Hodzic, 2024; Wright, 2019). While not an assurance of robust and quality research, reflexivity (defined as thoughtful self-awareness of an experience while living that experience; Finlay, 2002) contributes to ongoing consideration and analysis of the researcher’s place within the research. This prompts the researcher to unpack their own assumptions about those involved in the research and what the betterment of individuals and groups may mean. Reflexivity in qualitative behavior analysis invites researchers to reflect on the ways that they hold and use their power within research. It allows the identification of, and responses to, inherent power structures that exist in research environments which can be (often unknowingly) replicated within the research. Traditional research positions researchers as the ‘dominant knowledge-seeking authority’ (Pritchett et al., 2022) and participants as ‘silent subservient targets’ (Fawcett, 1991), creating the potential for ‘coercive cycles’ that involve control and counter control on the part of the researcher (Pritchett et al., 2022). While the potential for exerting control is not limited to quantitative investigations, reflecting on power imbalances within qualitative behavior analysis could allow researchers to revise and shape their behavior to move away from an ‘unconscious’ positioning as deciders, to ‘self-conscious’ researchers who interrogate the role they play in challenging the ‘broader cultural context’ (Ruiz, 1995, p. 174) through their research.

## A Call to What? Next Steps in Qualitative Behavior Analysis

The suggestion that qualitative behavior analysis could exist as a distinct, but related, branch of behavior analysis is novel. Necessarily, the scope of this argument and the potential implications of such an approach require rigorous attention and careful consideration from within the field. As a first step, this call to action is developed to stimulate discussion (and possible debate) on the merits of such a position, from behavior analysts themselves.

Accepting that a distinct branch of qualitative behavior analysis could have value (or, contact reinforcement) for the field leads to another set of questions: How do behavior analysts *do* qualitative behavior analysis? What skills do we need to acquire (and eventually master) to genuinely incorporate qualitative behavior analysis? Although by no means complete, the following suggestions are offered, as ways to move forward with a commitment to embracing qualitative behavior analysis. Critically, while these ideas are novel in the context of qualitative behavior analysis as a subdiscipline of behavior science, they are grounded in the foundational literature of behavior analysis (and thus consistent with the science as a whole; see Michael, 1993; Sidman, 1960; Skinner, 1974).

### Thinking Flexibly About the Best Research Approach for the Task

Rather than defaulting to small-n design as the methodological tool for any research question based in behavior analysis (Kazdin, 2021; Smith & Little, 2018), a qualitative behavior analyst would first consider the question they hope to answer, before selecting methods that best support this aim. In espousing the value of humble behaviorism, Neuringer described that the selection of a research approach ‘based on methodological consistency…may be less effective than selection based upon the questions’ (1991, p. 6), advocating for analysts to expand their research approaches to allow investigation of myriad important questions for behavior analysts. This may require analysts to interrogate the relational frames they hold around what ‘good quality’ research comprises, and if these frames are in service of developing ways to explore necessary and useful topics. In service of practicing flexibility when selecting methodological options, analysts may ask ‘which approaches will best generate insight into the phenomena I am investigating?’ and ‘how can I develop my skills in utilising those approaches?’ as part of qualitative behavior analysis.

***What behaviors are needed?*** Thinking flexibly about research approaches will require behavior analysts to build their knowledge of qualitative methods, to develop confidence in designing and conducting qualitatively derived studies which are coherent and robust (J. A. Smith, 2015). Aligned with an expanded call toward humble behaviorism (Kirby et al., 2022), this may include: reading broadly within qualitative literature to identify salient examples, attending conferences disseminating qualitative research in areas relevant to behavior analysts, and engaging with researchers who are familiar with qualitative approaches within, and likely outside, the field of behavior analysis. Such exposure to sound models of qualitative research may help to build knowledge and skills of qualitative behavior analysts. This step, if taken with care, will likely bolster the quality of qualitative inquiry in behavior analysis, such that our research is judged to be robust when viewed by experts outside the field.

### Being Open to Constructive Insights From Stakeholders

Aligned with a move toward compassionate behavior analysis (Marchese & Weiss, 2023; Taylor et al., 2018), qualitative behavior analysis extends an opportunity to canvas the experiences and perspectives of others (with distinct learning histories and private events to those of the researcher/analyst) toward positioning our research and practice with the wants and needs of stakeholders. Rather than taking a defensive position against critiques from within and outside our field (Critchfield, 2023), qualitative behavior analysis offers a way to better attend to and highlight the perspectives of others, in service of better positioning the social validity of behavior analytic research. Penney et al. (2023) describe that the field of behavior analysis ‘must be willing to own criticism and learn from it’, reminding us that stakeholder feedback serves a function, and gathering information around this behavior and likely function will help the field to address issues which contributed to such contingencies (Kelly et al., 2021). In qualitative behavior analysis, questions such as ‘which stakeholders can offer us novel information to further our practice?’ and ‘how can insights from outside of our field shape responding within the field toward our stated aims?’ could prompt responding.

***What behaviors are needed?*** The practice of listening inherent in qualitative research, for example, in allowing others to set the parameters of the investigation, highlighting other voices in the process and products, and applying an inductive (vs. deductive or researcher-driven) approach to analysis, may help to centre stakeholders in behavior analytic research (Pritchett et al., 2022), and generate insights from which analysts can shape their own behavior. Necessarily this involves qualitative behavior analysist honing our skills around accepting criticism without defensiveness, and willingness to change our own behavior in response to qualitative behavior analytic investigations (Dettmering & Hodzic, 2024; Penney et al., 2023). Such skills could be fostered through accessing literature, formal teaching, and professional development, which exemplify the range of qualitative lenses that could support stakeholder voice (Busch et al., 2023). These skills could also be fostered by accessing models for collaborative and co-constructed research (see Mathur et al., 2024, for a discussion around neurodiverse affirming research in behavior analysis), to broaden awareness of, and skills in developing, qualitative research which centres stakeholders.

### Engaging in Collaborative Research Endeavours

Given that qualitative behavior analysis is an emerging direction for behavior analysts and accepting that qualitative methods are well established in other fields, analysts should look to interdisciplinary research approaches in order to develop skills in qualitative inquiry. Many disciplines have a comprehensive grounding in qualitative research, which far exceeds that available to behavior analysts. Further, many fields with related aims or values to that of behavior analysis (for example, occupational therapy; D’Arrigo et al., 2020) have a long learning history of utilising qualitative research, and have developed structures (e.g., literatures, handbooks, training, supervision systems) to build the skill sets of their qualitative researchers. Behavior analysts need not ‘reinvent the wheel’. Instead, our field can foster skills in interdisciplinary working and collaboration, to develop our own capabilities in qualitative behavior analysis. Analysts may consider ‘who are the expert models, and how can I collaborate with them?’ in positioning their qualitative behavior analytic investigations as rigorous and robust.

***What behaviors are needed?*** Qualitative behavior analysts will benefit from collaborating with those from other disciplines who are champions in qualitative approaches, rather than forging a path alone. This step requires behavior analysts to practice and shape their interdisciplinary working and collaborative skills aligned with calls for better co-working in applied (Coy et al., 2024) and in research (Elcoro et al., 2023) contexts. Critically this involves accepting that philosophical positioning, alongside use of language and terminology (Becirevic et al., 2016), differs across disciplines. A willingness to accept such differences in service of collaboration is necessary for analysts interested in developing qualitative understandings. Specifically, analysts could focus on developing interpersonal collaboration skills (as described by Coy et al., 2024), and cross-disciplinary research skills (outlined by Elcoro et al., 2023) as first steps toward interdisciplinary research, to progress qualitative behavior analysis.

## Conclusion

This article presents the germination of qualitative behavior analysis as a distinct approach within behavior analysis. It represents a starting point to consider how qualitative behavior analysis can sit within behaviorism, complement established behavior analytic research approaches, and move the field further in the direction of equity and social justice commitments. This is a starting point; an invitation for the field to generate discussion around the place of qualitative approaches in behavior analysis, which could help to move the field in valued directions at an arguably critical time. More investigation is required to shape qualitative behavior analysis as a branch of behavior analysis, including interrogating how proposed tenets discriminate qualitative behavior analysis from other behavioral approaches, and identifying which behaviors analysts can engage in toward developing qualitative skillsets. It is hoped that this call echoes in spaces where a qualitative behavior analytic lens is valued, and where collaborative efforts can further nurture this seed.

## Data Availability

The manuscript has no associated data.
